# Tumor-Acidity Responsive Polymeric Nanoparticles for Targeting Delivery of Angiogenesis Inhibitor for Enhanced Antitumor Efficacy With Decreased Toxicity

**DOI:** 10.3389/fbioe.2021.664051

**Published:** 2021-03-24

**Authors:** Xiufeng Cong, Jun Chen, Ran Xu

**Affiliations:** ^1^Department of Oncology, Shengjing Hospital of China Medical University, Shenyang, China; ^2^Department of Thoracic Surgery, Shengjing Hospital of China Medical University, Shenyang, China

**Keywords:** anlotinib, polymeric nanoparticles, pH-responsiveness, side effect, lung cancer

## Abstract

Various nanocarriers with tumor targeting ability and improved pharmacokinetic property have been extensively utilized to reduce the toxicity of existing clinical chemotherapeutics. Herein, we showed that by encapsulating angiogenesis inhibitor anlotinib into polymeric nanoparticles, we could significantly decrease its *in vivo* toxicity. The introduction of pH-responsiveness into the nanocarrier further enhanced its anti-tumor activity. Systemic administration of the anlotinib-loaded nanocarrier into mice bearing A549 and 4T1 subcutaneous tumor received a higher tumor growth suppression and metastasis inhibition without detectable side effects. This strategy offers a promising option to improve the patient compliance of anlotinib.

## Introduction

Anlotinib, a small molecule multi-targeting tyrosine kinase inhibitor with a broad spectrum of inhibitory effects on tumor angiogenesis and growth by targeting vascular endothelial growth factor receptor (VEGFR), fibroblast growth factor receptor (FGFR), platelet-derived growth factor receptors (PDGFR), c-Kit, and so on (Chi et al., 2018; Zhong et al., 2018; Liu et al., 2020), has been widely used in clinical cancer therapy and can significantly prolong both progression-free survival (PFS) and overall survival (OS) in patients with advanced non-small-cell lung cancer (NSCLC) (Syed, 2018; Zhou et al., 2019). In addition, anlotinib also shows significant anti-tumor activity in various tumor types such as osteosarcoma (Wang et al., 2019), medullary thyroid carcinoma (Sun et al., 2018), liver cancer (Lin et al., 2018), and soft-tissue sarcoma (Chi et al., 2018). However, in our clinical observation, multifaceted toxicities and side effects appeared after oral anlotinib administration, e.g., liver damage, hypertension, diarrhea, hypothyroidism, and bleeding (Sun et al., 2016; Shao et al., 2019). How to improve the patient compliance of anlotinib is still an urgent clinical challenge and will bring benefits to more cancer patients.

Holding great potentials to minimize the side effects and improve the bioavailability of existing chemotherapeutic drugs in clinic, nanomedicines have been extensively applied for cancer theranostics (Pelaz et al., 2017; Tran et al., 2017). Several nanomedicines have been approved by FDA for clinical application such as Doxil (Barenholz, 2012) and Abraxane (Green et al., 2006) and shows enhanced anti-tumor activity with decreased side effects. Besides, in preclinical studies, various tumor microenvironment-responsive (Uthaman et al., 2018; Huang et al., 2020) or tumor targeting nanomedicines (Arranja et al., 2017; Dai et al., 2017) have also been developed and show superiorities than non-functional nanomedicines in antitumor activity and selectivity. Therefore, nanotechnology has great possibility to drug the highly toxic molecules or improve the benefits patients obtained from existing drugs (Barenholz, 2012). The unbalance between rapid tumor proliferation and dysfunctional blood vessel regeneration leads to the specific characteristics (e.g., acidic pH, hypoxia and over-expressed specific enzymes) of tumor microenvironment which can be reasonably utilized for the design of multifunctional nanomedicines (Fukumura and Jain, 2007; Whiteside, 2008; Ji et al., 2013).

In current study, to improve the anti-tumor activity and decrease the side effects of anlotinib, a tumor-acidity responsive polymeric nanocarrier loaded with anlotinib hydrochloride was developed. During the synthetic process, a pH-responsive polymer material PEOz-PLGA was introduced into the structure of nanoparticles and endowed the nanoparticles with pH-responsive property (Wang et al., 2017). The resulting NPs showed a typical core-shell structure with an average hydrous size of 170.2 nm. Both the *in vitro* drug release investigation and cell viability assay indicated PEOz-NP-A could rapidly release the encapsulated drugs under low pH condition and exhibited enhanced tumor cell killing efficacy. After administrated into tumor-bearing mice, PEOz-NP-A could significantly inhibit tumor growth without bringing obvious side effects in comparison with corresponding control groups. This pH-responsive polymeric nanocarrier offers a potential option to optimize the patient compliance of anlotinib in clinical usage.

## Results and Discussion

### Characterization of NPs

**FIGURE 1:**
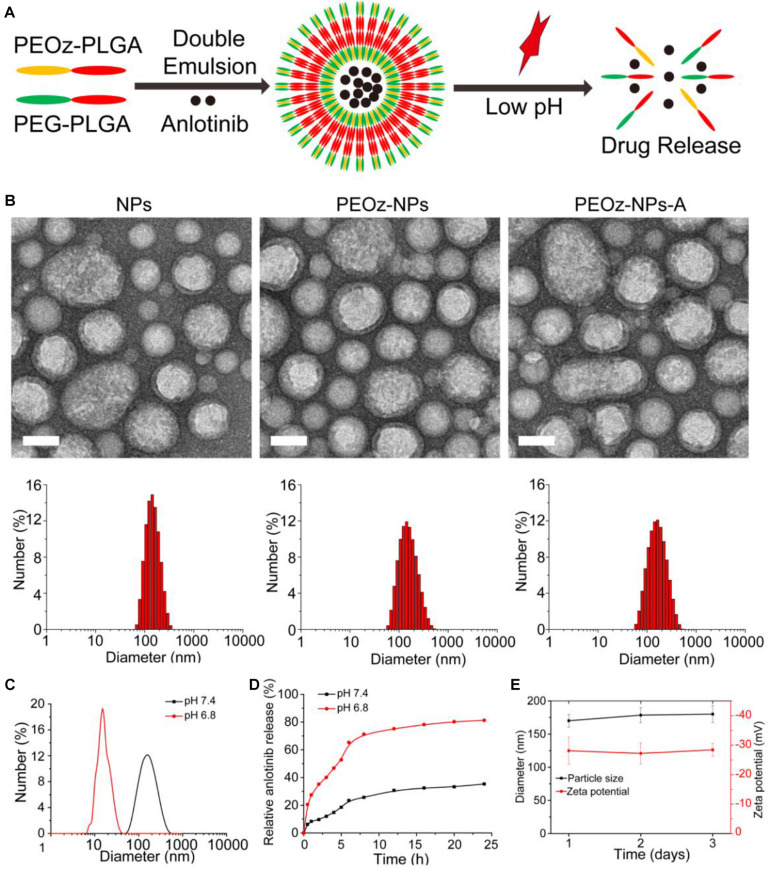
Construction and characterization of PEOz-NPs-A. **(A)** Schematic preparation of pH-responsive nanoparticle PEOz-NPs-A. **(B)** TEM imaging and DLS analysis of NPs, PEOz-NPs, and PEOz-NPs-A, Scale bars are 100 nm. **(C)** Size distribution of PEOz-NPs-A at different pH conditions (pH = 6.8, pH = 7.4). **(D)** Anlotinib release profile of PEOz-NPs-A at different pH conditions (pH = 6.8, pH = 7.4). **(E)** Stability evaluation of PEOz-NPs-A during 3 days storage by DLS analysis.

The biocompatible polymer PEG-PLGA was used to construct the anlotinib-loaded nanoparticles (NPs-A) by double emulsion method reported previously. To achieve pH-responsiveness, PEOz-PLGA, a pH-sensitive polymer material, was mixed into the PEG-PLGA solution during the preparation process to form pH-responsive nanoparticles (PEOz-NPs-A) (Figure 1A). We first optimized the drug loading efficacy using different ratios of polymeric materials and drugs (wt:wt). When the encapsulation efficiency (EE) increased to 32.4% at ratio of 20:5 which was selected for the subsequent experiments, the loading efficiency (LE) reached to its maximum 7.49% (Supplementary Table 1). Dynamic light scattering (DLS) characterization revealed the hydrodynamic diameter and zeta potential of PEOz-NPs-A were about ∼170.2 nm and −28.1eV, respectively (Figure 1B). The physicochemical properties of related drug formulations used in this study were also characterized (Supplementary Table 2). Transmission electron microscope (TEM) was used to characterize the morphology of nanoparticles. The results showed that PEOz-NPs-A exhibited a typical core-shell structure (Figure 1B). We next evaluated the responsiveness of PEOz-NPs-A nanoparticles to the acidic environment by DLS analysis and drug release investigation. The diameter of PEOz-NPs-A changed to 16.3 nm from 170.2 nm when the pH changed to 6.8 from 7.4 (Figure 1C). The results of drug release indicated that ∼50% anlotinib was released from PEOz-NPs-A after 5 h incubation in PBS at pH 6.8, while just ∼20% anlotinib was released from PEOz-NPs-A at pH 7.4. The total drug release of PEOz-NPs-A reached to ∼81% at pH 6.8 after 24 h incubation while just 35% at pH 7.4 (Figure 1D). Both of these results demonstrated that PEOz-NPs-A dissociated in a weakly acidic environment and rapidly released their loaded cargo in a pH-dependent manner. Finally, the stability of PEOz-NPs-A was also evaluated by DLS. We did not observe obvious changes of size and zeta potential during the 3 days storage (Figure 1E).

### *In vitro* Cell Internalization and Cytotoxicity

**FIGURE 2:**
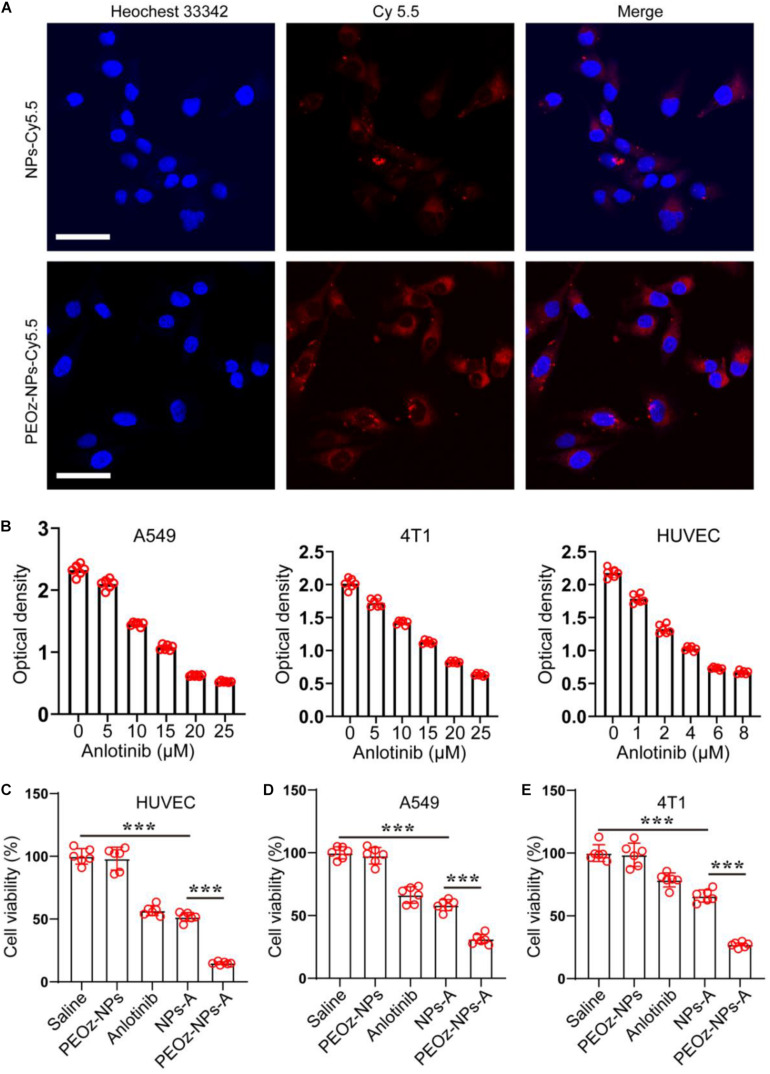
*In vitro* cell internalization and cytotoxicity of PEOz-NPs-A. **(A)** Confocal images of A549 cells after 8 h incubation with PEOz-NPs-A and NPs-A. The nanoparticles were labeled by fluorescent dye Cy5.5. Cell nuclei were stained with Hoechst 33342. The scale bar is 50 μm. **(B)** IC50 assay of anlotinib in three cell lines. **(C–E)** Cell viability of HUVEC **(C)**, A549 **(D)**, and 4T1 **(E)** treated with PBS, PEOz-NPs, anlotinib, NPs-A and PEOz-NPs-A for 24 h. (*n* = 6). ***P < 0.001.

We next studied the endocytosis of PEOz-NPs-A and NPs-A by Confocal microscopy. Both nanoparticles (NPs and PEOz-NPs) were labeled with fluorescent dye Cy5.5. After incubation for 8 h, significantly stronger fluorescent signals were observed within A549 tumor cells treated with PEOz-NPs-Cy5.5 than group treated with NPs-Cy5.5 (Figure 2A). The reason might be attributed to the rapid dye release of PEOz-NPs-Cy5.5 in the lysosome. Then, the IC50 of anlotinib hydrochloride for A549 human non-small cell lung cancer cells, 4T1 mouse mammary tumor cells and human umbilical vein endothelial cells (HUVEC) were first evaluated by CCK-8 assay. The results showed the IC50 for A549, 4T1, and HUVEC was 14, 15, and 4 μmol, respectively, and the HUVEC was more sensitive to anlotinib than other two cell lines (Figure 2B). To further investigate the *in vitro* tumor cell killing efficacy, A549, 4T1, and HUVEC were treated with different drug formulations for 24 h. Subsequently, the cell viability was measured by CCK-8 assay. The pH-responsive PEOz-NPs-A showed the maximal cytotoxicity than free anlotinib and NPs-A in three cell lines, indicating successful incorporation of PEOz-PLGA. The blank nanoparticles PEOz-NPs did not show any cytotoxicity, indicating its good biocompatibility *in vitro* (Figures 2C–E).

### *In vivo* Bio-Distribution of PEOz-NPs-A

**FIGURE 3:**
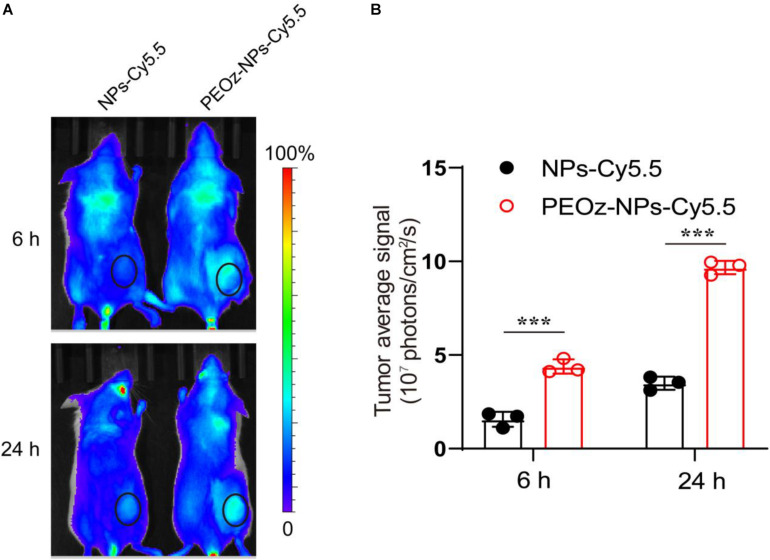
*In vivo* fluorescence imaging. **(A)** *In vivo* fluorescence images of BALB/c mice after administration of NPs-Cy5.5 and PEOz-NPs-Cy5.5 for 6 and 24 h. The circles indicate tumor region. **(B)** Quantification of tumor fluorescence intensity in A. (*n* = 3). ***P < 0.001.

We next investigated the bio-distribution of Cy5.5-labelled PEOz-NPs in BALB/c mice bearing subcutaneous 4T1 tumors. After intravenous injection of PEOz-NPs-Cy5.5 and NPs-Cy5.5 for 6 and 24 h respectively, the *in vivo* fluorescence signal was obtained by optical imaging system (IVIS Spectrum, PerkinElmer). As shown in Figure 3, a strong fluorescence signal was observed in the tumor area of mice treated with PEOz-NPs-Cy5.5 post-injection for 6 h, while no obvious signal was detected in tumor of NPs-Cy5.5 treated group. 24 h later, PEOz-NPs-Cy5.5 showed a further enhanced tumor accumulation. However, the NPs-Cy5.5 just showed a weak tumor accumulation post-injection for 24 h (Figures 3A,B). These results demonstrated that PEOz-NPs-Cy5.5 could significantly accumulate in tumor tissue and laid foundation for *in vivo* anti-tumor activity.

### *In vivo* Antitumor Activity of PEOz-NPs-A

**FIGURE 4:**
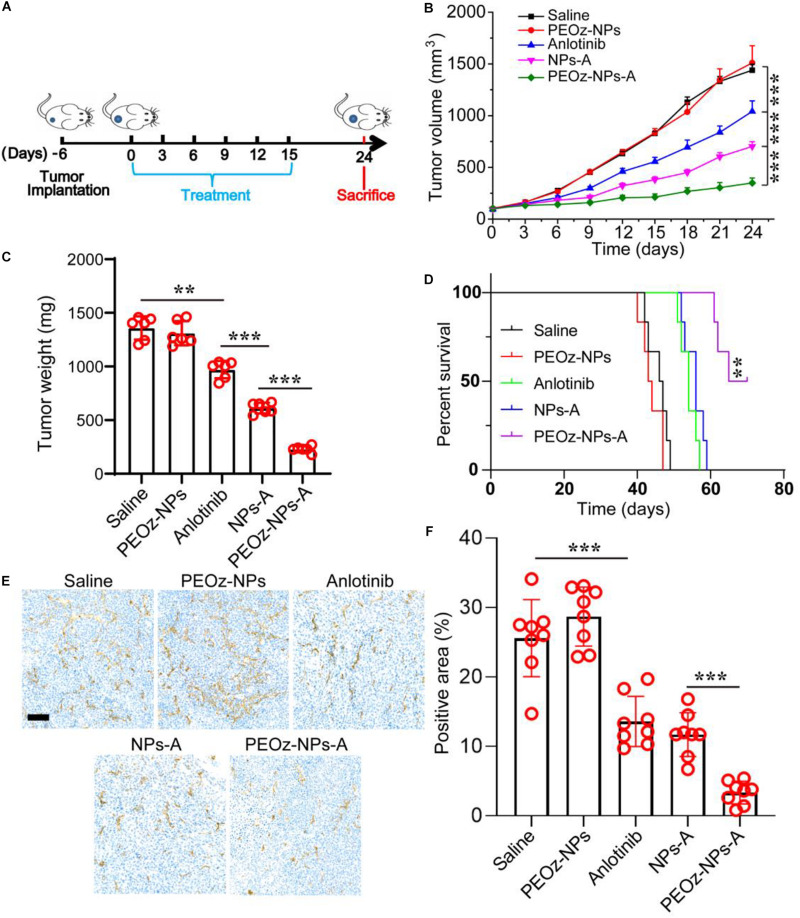
*In vivo* anticancer activity of PEOz-NPs-A at A549 tumor bearing mice. **(A)** Diagrammatic representation of the therapeutic procedure. **(B)** Tumor growth curves of 4T1 tumor-bearing BALB/c mice treated with different drug formulations including Saline, PEOz-NPs, Anlotinib, NPs-A and PEOz-NPs-A (*n* = 6). **(C)** Tumor weight of different treatment groups (*n* = 6). **(D)** Cumulative survival of the tumor-bearing mice treated with different drug formulations including Saline, PEOz-NPs, Anlotinib, NPs-A and PEOz-NPs-A. (*n* = 6). **(E)** Immunohistochemical staining of tumor tissue for vasculature marker CD 31. The scale bar is 100 m. **(F)** Tumor blood vessels in panel **(E)** was quantified. **P < 0.01 and ***P < 0.001.

We next examined the therapeutic efficacy of PEOz-NPs-A in an established A549 tumor model. When the tumor volume grown to approximately 100 mm^3^, the mice were randomly divided into five groups and intravenously injected with Saline, PEOz-NPs, Anlotinib, NPs-A, and PEOz-NPs-A respectively every 3 days for total six times (Figure 4A). After six injections, the tumor growth curve showed that PEOz-NPs-A treated group had a minimum average tumor volume compared with other treated groups. Compared with free Anlotinib-treated group, NPs-A treatment showed a significant stronger tumor inhibition due to the EPR effect of nanoparticles (Figure 4B). The tumor weight also showed the consistent results with tumor growth curve (Figure 4C). Mice receiving PEOz-NPs-A had a median survival time of 60 days, while mice receiving Saline, PEOz-NPs, Anlotinib, NPs-A just had median survival times of 30, 31, 42, and 45 days, respectively (Figure 4D). These results demonstrated that PEOz-NPs-A with pH-responsive feature could significantly enhance the antitumor effects. To examine the neovascularization inhibitory effect of different drug formulations *in vivo*, the vasculature marker CD31 in tumor tissue of various treatment groups was detected by immumohistochemical staining. As shown in Figures 4E,F, PEOz-NPs-A showed the highest antiangiogenic activity in comparison with Saline, PEOz-NPs, Anlotinib, and NPs-A groups.

**FIGURE 5:**
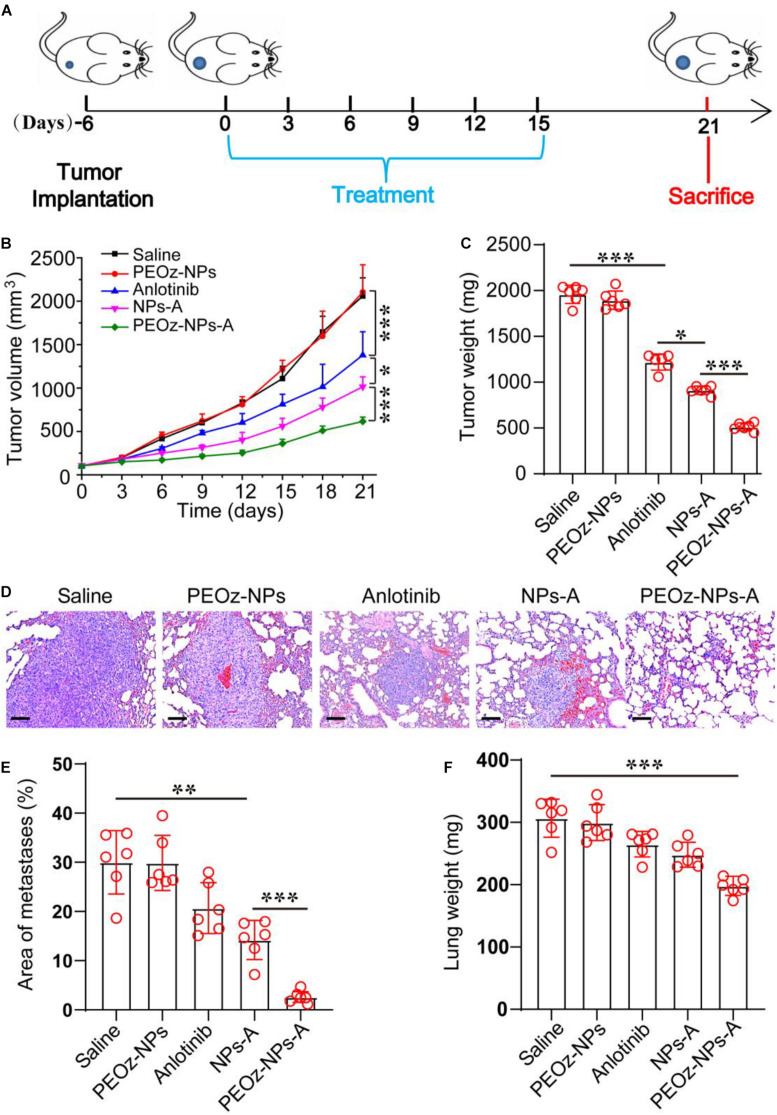
*In vivo* antitumor activity of PEOz-NPs-A at 4T1 tumor bearing mice. **(A)** Diagrammatic representation of the therapeutic procedure. **(B)** Tumor growth curves of 4T1 tumor-bearing BALB/c nude mice treated with different drug formulations including Saline, PEOz-NPs, Anlotinib, NPs-A and PEOz-NPs-A (*n* = 6). **(C)** Tumor weight of different treatment groups (*n* = 6). **(D)** H&E staining of lung tissue of mice treated with different drug formulations. The hyperchromatic region is tumor metastasis. The scale bars are 100 μm. **(E)** Quantification of metastatic area of lung tissues from mice treated with different drug formulations including Saline, PEOz-NPs, Anlotinib, NPs-A and PEOz-NPs-A. (*n* = 10). **(F)** Lung weight from animals in the treatment groups. (*n* = 6). *P < 0.05, **P < 0.01, and ***P < 0.001.

To explore whether the inhibition of angiogenesis had effects on tumor metastasis, we established a subcutaneous 4T1 breast tumor mouse model, which preferred to metastasize to the lung tissue. Similar to the results obtained in A549 tumor model, PEOz-NPs-A showed the highest tumor growth inhibition after six drug administrations (Figures 5A–C). After 30 days from tumor inoculation, the mice were sacrificed and metastatic foci on the surface of lungs were analyzed by H&E staining. Hematoxylin and eosin (H&E) staining of the lungs revealed that 32.8% area of lung tissue was occupied by metastasis in the control group. In contrast, PEOz-NPs-A treated group just had 2.5% occupied lung tissue, outperforming other drug formulations treated groups in reducing lung metastasis (Figures 5D,E). The lung weight of mice receiving PEOz-NPs-A was also the lowest (Figure 5F). These observations indicated PEOz-NPs-A treatment could also remarkably inhibit tumor metastasis.

### Safety Evaluation

**FIGURE 6:**
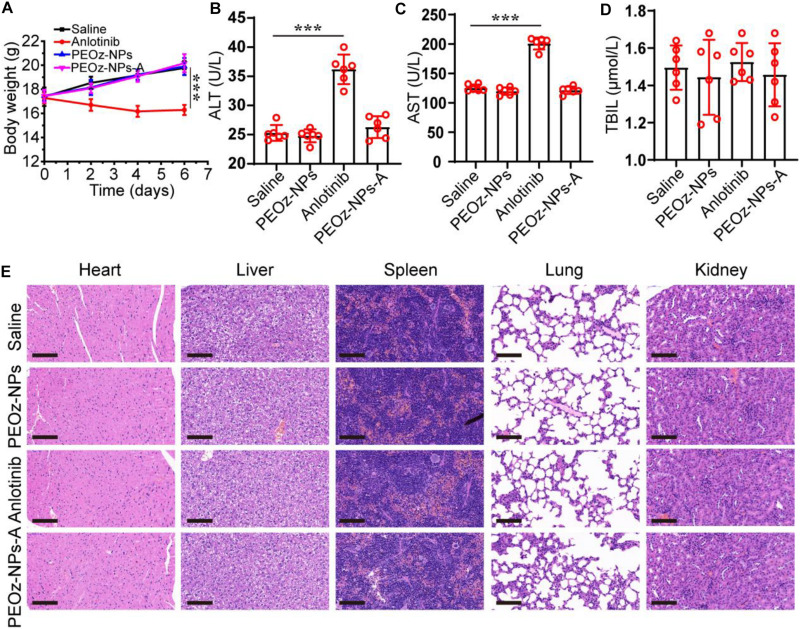
*In vivo* safety evaluation of PEOz-NPs-A. **(A)** Body weigh changes of mice treated with Saline, PEOz-NPs, Anlotinib, and PEOz-NPs-A (*n* = 6). Biochemical indicators of liver including ALT **(B)**, AST **(C)**, and TBIL **(D)**. (*n* = 6). **(E)** H&E staining of major organs isolated from mice treated with Saline, PEOz-NPs, Anlotinib, and PEOz-NPs-A. The scale bars are 100 μm. ***P < 0.001.

Finally, we investigated the *in vivo* biocompatibility of PEOz-NPs-A on major organs, especially liver. The normal mice were divided into four treatment groups: Saline, PEOz-NPs, Anlotinib, and PEOz-NPs-A. The body weight was also recorded every 2 days. After three times treatment within 1 week, the mice were killed and the blood serum was collected for biochemical analysis. Meanwhile, the major organs were also isolated and used for H&E staining. Only the body weight of mice treated with free Anlotinib had a significant decrease (Figure 6A). Serum biochemical indicator of liver (ALT and AST) reflected that free Anlotinib had an obvious liver damage. When Anlotinib was encapsulated into nanoparticles, the liver damage was significantly alleviated (Figure 6B–D). We did not observe any histological changes in current study in all treated groups (Figure 6E). These results suggest that the side effects of Anlotinib were greatly decreased by encapsulating it into nanoparticles.

## Conclusion

In summary, we developed a pH-responsive polymeric nanocarrier PEOz-NPs-A for targeting delivery of angiogenesis inhibitor anlotinib and local drug release to realize the enhancement of anti-tumor activity with minimum sided effects. The *in vitro* drug release profile and cell viability assay demonstrated the successful construction of the nanocarrier. *In vivo* anti-tumor experiments exhibited significantly improved tumor suppression and metastasis inhibition. One potential mechanism of metastasis inhibition was the reduction of primary tumor, leading to the diverse tumor size in different treatment groups. In addition, many studies indicated anlotinib could decrease metastasis by inhibiting the lymphangiogenesis *via* VEGFR 3 signaling pathway (Garcia-Caballero et al., 2017). Compared with free anlotinib, the established PEOz-NPs-A presented better *in vivo* biosecurity. These results make the PEOz-NPs-A particularly attractive candidate strategy for targeting delivery of anlotinib to tumor tissue and decreasing the toxicity of anlotinib. In this study, to ensure the biosafety and large-scale production, we chose the FDA-approved material PEG-PLAG as the basic block to construct the anlotinib-loaded nanomedicine, providing convenience for further clinical transformation. However, there are lots of further works needed to verify its effectiveness in clinic usage. For example, the action mechanism of anlotinib-loaded nanomedicine should be investigated in detail. In the only previous study, Zhang’ group developed a GSH-responsive anlotinib-loaded nanomicelle decorated with a cyclic RGD peptide on the surface (Zhang et al., 2020). Differing from their work, our anlotinib-loaded nanomedicine could release drugs in the tumor microenvironment and acted on both tumor cells and tumor endothelial cells. For the anti-angiogenic nanomedicine design, the payload should be released in tumor microenvironment to ensure its efficacy.

## Materials and Methods

### Materials

Monomethoxy poly (ethylene glycol) (mPEG)-poly-(lactic-co-glycolic acid) (PLGA; molar ratio of D,L-lactic to glycolic acid, 75:25) was purchased from Jinan Daigang Biotechnology Co., Ltd. (Jinan, China). Poly (2-ethyl-2-oxazoline) (PEOz)-poly-(lactic-co-glycolic acid) (PLGA; molar ratio of D,L-lactic to glycolic acid, 75:25) was purchased from Xian Ruixi Biological Technology Co., Ltd. (Xian, China). Anlotinib Hydrochloride was kindly supplied by Chia Tai Tianqing Pharmaceutical Group Co., Ltd. (Jiangsu, China). Cy 5.5 (S1061) was purchased from Solarbio Life Science. Anti-CD31 antibody was purchased from Abcam (cat. no. ab182981) (Cambridge, MA, United States). CCK-8 (cat. no. CK04) was purchased from DOJINDO.

### Methods

#### Preparation of Nanoparticles

PEG-PLGA nanoparticles were synthesized by the double emulsion method reported in the previous published paper (Wang et al., 2013). Briefly, 20 mg mPEG-PLGA dissolved in 1 mL dichloromethane and 200 μL deionized water or 200 μL Anlotinib hydrochloride solution or 200 μL Cy5.5 dye solution were mixed together and added into a 10 mL centrifuge tube. The mixture was emulsified by sonication *via* a probe ultrasonicator at 30% power for 5 min within ice-bath. Then, 2 mL 5% sodium cholate was added into the tube. The mixture was emulsified again by sonication at 35% power for another 5 min. Finally, the mixture was added dropwise into 10 mL 0.5% sodium cholate and stirred for 10 min. The dichloromethane in the solution was moved out by vacuum evaporation and the nanoparticles were collected by centrifugation at 15,000 *g* for 30 min and resuspended in 1 mL deionized water for further usage. For the preparation of pH-responsive nanoparticles (PEOz-NPs-A), 20 mg mPEG-PLGA was replaced by 18 mg mPEG-PLGA and 2 mg PEOz-PLGA during the above procedure.

#### Nanoparticle Characterization

The particle size and zeta potential of the prepared nanoparticles were measured by DLS at a concentration of 1 mg/mL. The morphology was observed by TEM. Briefly, 10 μL nanoparticle solution with a concentration of 0.1 mg/ml was dropped onto the copper grids. After incubation for 5 min, the solution was moved out by liquid-moving machine. Then, 8 μL phosphotungstic acid staining solution was used to stain the sample and TEM images were carried out.

For the drug encapsulation evaluation, the obtained nanoparticles were demulsified by acetonitrile and the amount of Anlotinib hydrochloride was detected by High Performance Liquid Chromatography at 220 nm. The following formulae were used to calculate the drug LE and drug EE:

EE = (weight of loaded drug)/(weight of initially added drug) × 100%;

LE = (weight of loaded drug in the nanoparticle)/(weight of the nanoparticle) × 100%.

#### *In vitro* Drug Release Profile

The above prepared pH-responsive nanoparticles (PEOz-NPs-A) were added into two dialysis bags and, respectively, transferred into two 50 mL centrifuge tubes with 30 mL of PBS at different pH values (6.8 and 7.2). The centrifuge tubes were placed in a shaker and incubated at 37°C for different time intervals. In each time point, 1 ml of PBS outside the lysis bag was taken out and 1 ml of fresh PBS at corresponding pH value was added. Finally, the amount of Anlotinib hydrochloride in the 1 ml of PBS was determined by High Performance Liquid Chromatography at 220 nm based on the previous report (Gao et al., 2020). Then, the accumulated drug release was calculated.

#### Cell Cultures

Human non-small cell lung cancer cell A549, and HUVEC were purchased from the American Type Culture Collection (ATCC, Rockville, MD, United States) and cultured at 37°C and 5% carbon dioxide in DMEM supplemented with 10% FBS and 1% penicillin and streptomycin. Mouse breast cancer cell 4T1 was purchased from the American Type Culture Collection (ATCC, Rockville MD, United States) and cultured at 37°C and 5% carbon dioxide in 1,640 RPMI supplemented with 10% FBS and 1% penicillin and streptomycin.

#### *In vitro* Cytotoxicity

For the cytotoxicity studies, A549, 4T1, and HUVEC were sub-cultured into 96-well plates at a density of 5 × 10^3^ cells per well for 24 h. Then, the tumor cells were treated with different drug formulations of PBS, PEOz-NPs, Anlotinib, NPs-A, and PEOz-NPs-A. The corresponding does of anlotinib were 10, 10, and 4 μmol for A549, 4T1, and HUVEC respectively. After 24 h incubation, the culture medium was replaced by fresh medium containing 10% CCK-8 assay buffer and incubation for another 1 h. The cell viability was detected by microplate reader at a wavelength of 450 nm.

#### Confocal Microscope

4T1 cells were sub-cultured into confocal petri dishes at a density of 1 × 10^5^ cells in each dish for 24 h. Then, the medium was replaced by fresh medium with Cy5.5-labelled nanoparticle NPs and pH-responsive nanoparticle Cy5.5-PEOz-NPs. After incubation for 8 h, the cellular nucleus was stained using Hoechst 33342 and the cells were observed by laser scanning Confocal microscope.

#### *In vivo* Targeting and Imaging

For the animal imaging, the tumor volume was allowed to grow to ∼200 mm^3^. The mice were divided into two groups and intravenously injected with 100 μL NPs-Cy5.5 and PEOz-NPs-Cy5.5. After injected for 6 and 24 h, the mice were imaged by an optical imaging system (IVIS Spectrum, PerkinElmer).

#### Animal Models and *in vivo* Studies

Female BALB/c nude mice and BALB/c mice, 6–8 weeks old and with an average body weight 18 g, were purchased from Beijing Vital River Laboratories. All animal experiments were undertaken according to the China Medical University Animal Care and Use Committee. The cultured A549 and 4T1 tumor cells were collected and suspended by a mixture of PBS and matrigel (1:1) and transplanted into the subcutaneous area on the dorsal flank of each mouse. When the tumor volume grown to 100 mm^3^ (volume = length × width^2^/2, measured with a Vernier caliper), the mice were then randomly divided into different five treated groups including Saline, PEOz-NPs, Anlotinib, NPs-A, and PEOz-NPs-A. The mice were intravenously injected with 100 μL solutions with different drug formulations (equivalent to 1 mg kg^–1^ bodyweight of Anlotinib) every 3 days for total six times. The tumor volumes of each mouse were measured with a Vernier caliper every 3 days.

#### Safety Evaluation

Female BALB/c mice, 6–8 weeks old and with an average body weight 18 g, were randomly divided into four treated groups including Saline, PEOz-NPs, Anlotinib, and PEOz-NPs-A. The mice were intravenously administrated with 100 μL drug solutions (equivalent to 2 mg kg^–1^ bodyweight of Anlotinib) every 2 days for total three times. Post-treatment for 7 days, the blood of mice were drawn from ocular venous plexus and the serum was separated by centrifugation at 3,000 rpm/min for 10 min. The major organs were also isolated for histologic analysis.

#### Statistical Analyses

Statistical analyses were performed using SPSS 19.0. Student’s *t*-test was used for comparison between two groups. One-way analysis of variance (ANOVA) followed by Tukey’s *post hoc* test was used for comparison of three or more groups. All the results are presented as means ± SD. *P* < 0.05 was considered statistically significant.

## Data Availability Statement

The original contributions presented in the study are included in the article/Supplementary Material, further inquiries can be directed to the corresponding authors.

## Ethics Statement

The animal study was reviewed and approved by the China Medical University Animal Care and Use Committee.

## Author Contributions

XC and RX conceived and designed the experiments and wrote the manuscript. XC and JC performed the experiments. All authors discussed the results and commented on the manuscript.

## Conflict of Interest

The authors declare that the research was conducted in the absence of any commercial or financial relationships that could be construed as a potential conflict of interest.

## Supplementary Material

Supplementary Table 1 Encapsulation efficiency and loading efficiency of Anlotinib in PEOz-NPs-A at various weight ratios of Polymer/Anlotinib. 20:5 was chosen in this study.

Supplementary Table 2 Characterization of different drug formulations by DLS.
