# Supplementary material for: Tumor-Acidity Responsive Polymeric Nanoparticles for Targeting Delivery of Angiogenesis Inhibitor for Enhanced Antitumor Efficacy With Decreased Toxicity
Source: Front Bioeng Biotechnol. 2021 Mar 24;9:664051. doi: 10.3389/fbioe.2021.664051 (PMC8024478; doi:10.3389/fbioe.2021.664051)
Supplement: Supplementary Table 1 — Encapsulation efficiency and loading efficiency of Anlotinib in PEOz-NPs-A at various weight ratios of Polymer/Anlotinib. 20:5 was chosen in this study. [file Table_1.docx]

**Supplementary Table 1. Encapsulation efficiency and loading efficiency of Anlotinib in PEOz-NPs-A at various weight ratios of Polymer/Anlotinib. 20:5 was chosen in this study.**

| Polymer : Anlotinib Ratio | 20：5 | 20：4 | 20：3 | 20：2 |
| --- | --- | --- | --- | --- |
| EE | 32.4% | 38.6% | 40.3% | 48.0% |
| DL | 7.49% | 7.17% | 5.70% | 4.58% |
