# Supplementary material for: Tumor-Acidity Responsive Polymeric Nanoparticles for Targeting Delivery of Angiogenesis Inhibitor for Enhanced Antitumor Efficacy With Decreased Toxicity
Source: Front Bioeng Biotechnol. 2021 Mar 24;9:664051. doi: 10.3389/fbioe.2021.664051 (PMC8024478; doi:10.3389/fbioe.2021.664051)
Supplement: Supplementary Table 2 — Characterization of different drug formulations by DLS. [file Table_2.docx]

**Supplementary Table 2. Characterization of different drug formulations by DLS.**

|  | NPs | NPs-A | PEOz-NPs | PEOz-NPs-A |
| --- | --- | --- | --- | --- |
| Size | 137.1 ± 10.2 | 142.8 ± 8.4 | 152.4 ± 12.4 | 170.2 ± 9.6 |
| PDI | 0.251 ± 0.06 | 0.208 ± 0.09 | 0.182 ± 0.05 | 0.198 ± 0.06 |
| Zeta | -25.4 ± 2.2 | -26.6 ± 3.4 | -27.3 ± 3.2 | -28.1 ± 4.6 |
